# Trends in gastric cancer burden in the Western Pacific region from 1990 to 2021 and projections to 2040

**DOI:** 10.3389/fonc.2025.1506479

**Published:** 2025-03-12

**Authors:** Tao Zhang, Yiqun Zhang, Xiaofei Leng

**Affiliations:** ^1^ Department of Gastric and Colorectal Surgery, General Surgery Center, The First Hospital of Jilin University, Changchun, Jilin, China; ^2^ Department of Gynecology, Taihe Hospital, Hubei University of Medicine, Shiyan, Hubei, China; ^3^ State Key Laboratory of Ultrasound in Medicine and Engineering, Chongqing Medical University, Chongqing, China; ^4^ Department of Gynecologic Oncology, Beijing Obstetrics and Gynecology Hospital, Capital Medical University, Beijing, China

**Keywords:** gastric cancer, GBD, western pacific region, incidence, mortality, DALYs

## Abstract

**Background:**

Gastric cancer (GC) is a major public health concern, particularly in the Western Pacific, a high-incidence region that bears significant economic and social burdens.

**Methods:**

Based on data from the Global Burden of Diseases, Injuries, and Risk Factors Study 2021, we conducted a comprehensive analysis of trends in the burden of GC in the Western Pacific from 1990 to 2021. We compared these trends with global and World Health Organization regional patterns, with a particular focus on geographic, gender, and age disparities. Health inequality was analyzed by comparing countries with different Socio-demographic Index (SDI) levels. Future trends in age-standardized rates were projected using the Bayesian Age-Period-Cohort (BAPC) model.

**Results:**

The GC burden of Western Pacific region remains above the global average, but improvements have outpaced global trends. China carries the highest burden, accounting for over half of regional cases, deaths, and disability-adjusted life years. While South Korea and Japan also experience high burdens, they have achieved notable reductions. Males consistently face higher burdens across age groups. Health inequality analysis shows narrowing gaps between high- and low-SDI countries, with the burden shifting toward less developed nations. BAPC model projections indicate a further decline in the GC burden by 2040.

**Conclusion:**

Despite substantial progress in countries like Japan and South Korea, continued focus is needed on less developed regions to reduce the remaining GC burden in the future.

## 1 Introduction

Gastric cancer (GC) is a widespread global health issue (1). According to the Global Cancer Statistics 2022, GC ranks as the fifth most common cancer globally and the fifth leading cause of cancer-related mortality, accounting for approximately 5% of all cancer cases and deaths (2). It poses a significant public health challenge worldwide, particularly in the Western Pacific region, where the burden of GC remains notably high (3). This region includes countries such as China and Japan, where the number of GC cases accounts for over half of the global total (4). Although radiotherapy, chemotherapy, and surgical treatments are widely used in cancer management, the insidious nature of GC often leads to late-stage diagnoses, resulting in persistently high mortality rates (5, 6). Therefore, studying the epidemiological trends of this disease is crucial for developing effective prevention and control strategies.

The global incidence and mortality rates of GC have shown a declining trend, largely attributed to the widespread use of antibiotics reducing Helicobacter pylori infection and improvements in food processing conditions (7–11). However, significant regional disparities in lifestyle factors, Helicobacter pylori prevalence, and the coverage of health screening programs have led to substantial differences in the burden of GC across countries and regions (12–14). To date, regional epidemiological studies on GC remain scarce. The Western Pacific region is of particular concern, as it accounts for 50% of the global GC cases, with South Korea, Japan, China, and Australia identified as high-incidence areas (3). Yet, there remains a paucity of research on the burden of GC in this region, highlighting the urgent need for further epidemiological studies to address this gap.

This study utilized data from the Global Burden of Diseases, Injuries, and Risk Factors Study (GBD) 2021, released in May 2024, which was one of the most comprehensive global disease burden studies to date (15–17). Our study represents the first study specifically focusing on the burden of GC in the Western Pacific region. Initially, the study analyzed the distribution characteristics and trends of GC incidence, mortality, and disability-adjusted life years (DALYs) in the Western Pacific region from 1990 to 2021. Subsequently, the Bayesian Age-Period-Cohort (BAPC) model was employed to project the GC age-standardized rates (ASRs) for 2040. The aim of this study was to provide scientific evidence for policymakers by exploring the distribution characteristics and future trends of GC burden in the Western Pacific region, thereby assisting in the development of more effective prevention and control strategies to reduce GC burden and improve patient survival rates.

## 2 Materials and methods

### 2.1 Study design

This study is a retrospective cross-sectional analysis based on GBD 2021, aimed at exploring the differences in and trends of GC incidence, mortality, and DALYs in the Western Pacific region from 1990 to 2021. Additionally, the BAPC model was used to predict ASRs for selected countries, extending to 2040. The data utilized in this study are publicly available and do not involve personal privacy or sensitive information. The original study received ethical approval; therefore, no additional ethical clearance is required for this research.

### 2.2 Data sources

In May 2024, the GBD project released its 2021 dataset, provided by the Institute for Health Metrics and Evaluation (16). Covering the period from 1990 to 2021, the dataset includes epidemiological assessments of 371 diseases and 88 risk factors across 204 countries and territories. It evaluates metrics such as incidence, prevalence, mortality, years lived with disability, years of life lost, and disability-adjusted life years (15, 17). Data sources include death registries, verbal autopsies, censuses, household surveys, disease registries, and healthcare utilization records (15, 17). The data is available at https://vizhub.healthdata.org/gbd-results/, with additional source information at https://ghdx.healthdata.org/gbd-2021/sources. Diseases are defined using International Classification of Diseases codes, and modeling tools like DisMod-MR and spatiotemporal Gaussian process regression are used for estimating cancer burden (15, 17, 18). The data process includes adjustments for heterogeneity and bias, with uncertainty analyzed through Monte Carlo simulation (15, 17). More details on the models can be found at https://www.healthdata.org/gbd/methods-appendices-2021/cancers.

### 2.3 Study region and country selection

The World Health Organization (WHO) divides the world into six regions: Africa, the Americas, Eastern Mediterranean, Europe, South-East Asia, and the Western Pacific (19). This study focuses on 31 countries and regions within the Western Pacific (hereafter referred to as countries) and compares them to global data (20). The 31 countries included are: Papua New Guinea, Solomon Islands, Vanuatu, Cambodia, Laos, Kiribati, Marshall Islands, Tuvalu, Micronesia, Samoa, Mongolia, Nauru, Tonga, Vietnam, the Philippines, Fiji, Tokelau, China, American Samoa, Niue, Malaysia, Palau, Northern Mariana Islands, Cook Islands, Guam, Brunei, Australia, New Zealand, Singapore, Japan, and South Korea. The study primarily analyzed the trends in GC incidence, mortality, and DALYs for these countries between 1990 and 2021.

### 2.4 Statistical analysis

#### 2.4.1 Trends in GC burden from 1990 to 2021

The percentage change in the number of incident GC cases from 1990 to 2021 in the Western Pacific region and globally was calculated using the following formula: (number of incident cases in 2021 – number of incident cases in 1990)/number of incident cases in 1990 (21). The same method was then applied to calculate the percentage change in the number of deaths and DALYs.

The study obtained ASRs for the total population across the Western Pacific region, globally, and for 31 countries within the Western Pacific from 1990 to 2021. The standard error (SE) was calculated using the following formula: SE = (upper – lower)/(1.96 × 2), where “upper” and “lower” represent the boundaries of the uncertainty interval (UI) for the ASRs obtained from GBD (22). Subsequently, a joinpoint regression analysis was conducted using the joinpoint Desktop software, provided by the National Cancer Institute, which can be accessed from their official website (https://surveillance.cancer.gov/joinpoint/) (23). The Permutation test model was applied in the joinpoint regression, and the average annual percent change (AAPC) and its 95% confidence intervals (CIs) were calculated using the Parametric Method.

#### 2.4.2 Health inequality analysis

To assess the inequality in GC burden across different income levels, data on GC burden for 204 countries and territories in 1990 and 2021 were collected, and the Slope Index of Inequality (SII) and Concentration Index were calculated (24). These are two standard indicators of absolute and relative gradient inequality, respectively, used to quantify the inequality in the distribution of GC burden across countries. SII is a measure of health inequality, primarily used to assess disparities in health outcomes across different socioeconomic groups. It is derived by ranking the population according to socioeconomic status and then calculating the slope of a linear regression for health outcomes along this ranking. The Concentration Index is an indicator commonly used to measure inequality in the distribution of income or wealth, and it can also be applied to the analysis of health outcomes. It reflects the degree of inequality by calculating the distribution of health outcomes across different socioeconomic strata.

#### 2.4.3 BAPC predictive model

According to the World Bank’s classification, countries were divided into three income levels: high-income, upper-middle-income, and lower-middle-income. In this study, two countries were selected from each income category (24). Island nations or geographically isolated countries were deliberately excluded due to the potential impact of unique healthcare systems and health technology factors on GC burden data. Japan and Singapore were selected to represent the high-income group, while China and Malaysia were chosen for the upper-middle-income group. Lastly, Vietnam and the Philippines were selected to represent the lower-middle-income category.

The BAPC model plays a crucial role in epidemiological research, helping to capture dynamic changes and their components. As a Bayesian statistical approach, BAPC models the independent effects of age, period, and birth cohort to analyze and predict demographic data (25). The core idea of Bayesian inference is to treat uncertain parameters as random variables with prior distributions while assuming that effects at adjacent time points are similar (26). Consequently, it is commonly assumed that the second-order differences of all time effects follow a normal distribution with a mean of zero, and a second-order random walk is used as a smoothing prior for age, period, and cohort effects to enhance model stability and reliability (26). This predictive model has been widely applied in the GBD research field (27–29). In this study, the BAPC model is employed to project ASRs for six countries up to 2040, with model fitting conducted using INLA (www.r-inla.org) and the BAPC R package (30–32).

The joinpoint analysis in this study was conducted using joinpoint software (5.2.0.0), while all other analyses were performed using R software (4.4.1) (33).

## 3 Result

### 3.1 GC burden in the Western Pacific compared to global and WHO regions

In 2021, the Western Pacific region reported 1,230,232.61 (95% UI: 1,052,350.05–1,409,969.66) new GC cases, accounting for 61.48% of the global total and ranking highest among WHO regions (**Table 1**). From 1990 to 2021, incident cases increased by 37.12%, surpassing the global average, while the improvement in incidence rates was second only to Europe (**Table 1**). The ASIR in 2021 was 26.76 (95% UI: 21.71–32.33) per 100,000 population, the highest globally, yet the region’s ASIR improvement from 1990 to 2021 exceeded the global average, second only to Europe (**Table 1**).

**Table 1:** Trends in incident cases and ASIR of GC globally and across 21 GBD regions from 1990 to 2021.

Location	Incident cases			ASIR		
	1990 (95% UI)	2021 (95% UI)	Percentage Change	1990 (95% UI)	2021 (95% UI)	AAPC (95% CI)
Global	980899.43 (891306.83–1072236.02)	1230232.61 (1052350.05–1409969.66)	25.42%	24.76 (22.58–27.00)	14.33 (12.23–16.41)	-1.77 (-1.91 to -1.63)
Western Pacific Region	551554.15 (476821.81–622213.73)	756302.53 (613600.98–917865.56)	37.12%	47.88 (41.93–54.09)	26.76 (21.71–32.33)	-1.88 (-1.99 to -1.76)
African Region	19965.13 (16250.83–22440.81)	33074.22 (26682.54–37943.25)	65.66%	8.91 (7.34–10.01)	6.55 (5.34–7.47)	-0.99 (-1.02 to -0.96)
Region of the Americas	79820.85 (76592.96–81987.31)	114876.50 (106042.61–122217.97)	43.92%	13.08 (12.53–13.44)	8.65 (7.99–9.20)	-1.30 (-1.40 to -1.21)
South-East Asia Region	66595.00 (56191.54–81055.78)	118433.76 (101971.95–139049.07)	77.84%	9.05 (7.69–11.19)	6.48 (5.57–7.59)	-1.06 (-1.22 to -0.90)
European Region	239409.06 (230619.93–245030.00)	165309.79 (154242.45–173820.30)	-30.95%	22.37 (21.53–22.90)	10.09 (9.47–10.60)	-2.52 (-2.67 to -2.38)
Eastern Mediterranean Region	18933.90 (14632.66–21463.94)	36340.33 (25798.41–41219.42)	91.93%	10.45 (8.09–11.84)	8.25 (5.79–9.30)	-0.75 (-0.89 to -0.61)

ASIR, age-standardized incidence rate; GC, gastric cancer; GBD, Global Burden of Diseases, Injuries, and Risk Factors Study; UI, uncertainty interval; AAPC, average annual percentage change; CI, confidence interval.

In 2021, GC deaths in the Western Pacific region reached 533,973.62 (95% UI: 431,189.38–647,568.07), accounting for 55.95% of the global total, the highest among WHO regions (**Supplementary Table 1**). Since 1990, deaths have risen faster than the global trend, yet the region’s improvement ranks second only to Europe (**Supplementary Table 1**). The ASMR in 2021 was 18.94 (95% UI: 15.30–22.87) per 100,000 population, the highest among WHO regions and well above the global average (**Supplementary Table 1**). Despite this, ASMR has declined significantly since 1990, with progress outpacing the global rate, second only to Europe (**Supplementary Table 1**).

In 2021, GC accounted for 12,332,045.27 (95% UI: 9,929,295.78–15,227,868.18) DALYs, making up 54.12% of the global total and the highest among WHO regions (**Supplementary Table 2**). From 1990 to 2021, DALYs declined by 4.76%, with improvements surpassing the global average, second only to Europe. The 2021 ASDR was 437.57 (95% UI: 352.93–539.40) per 100,000 population, the highest among WHO regions and well above the global average (**Supplementary Table 2**). Since 1990, ASDR has declined, with progress outpacing global trends, second only to Europe (**Supplementary Table 2**).

### 3.2 GC burden in countries and territories of the Western Pacific

**Figure 1:**
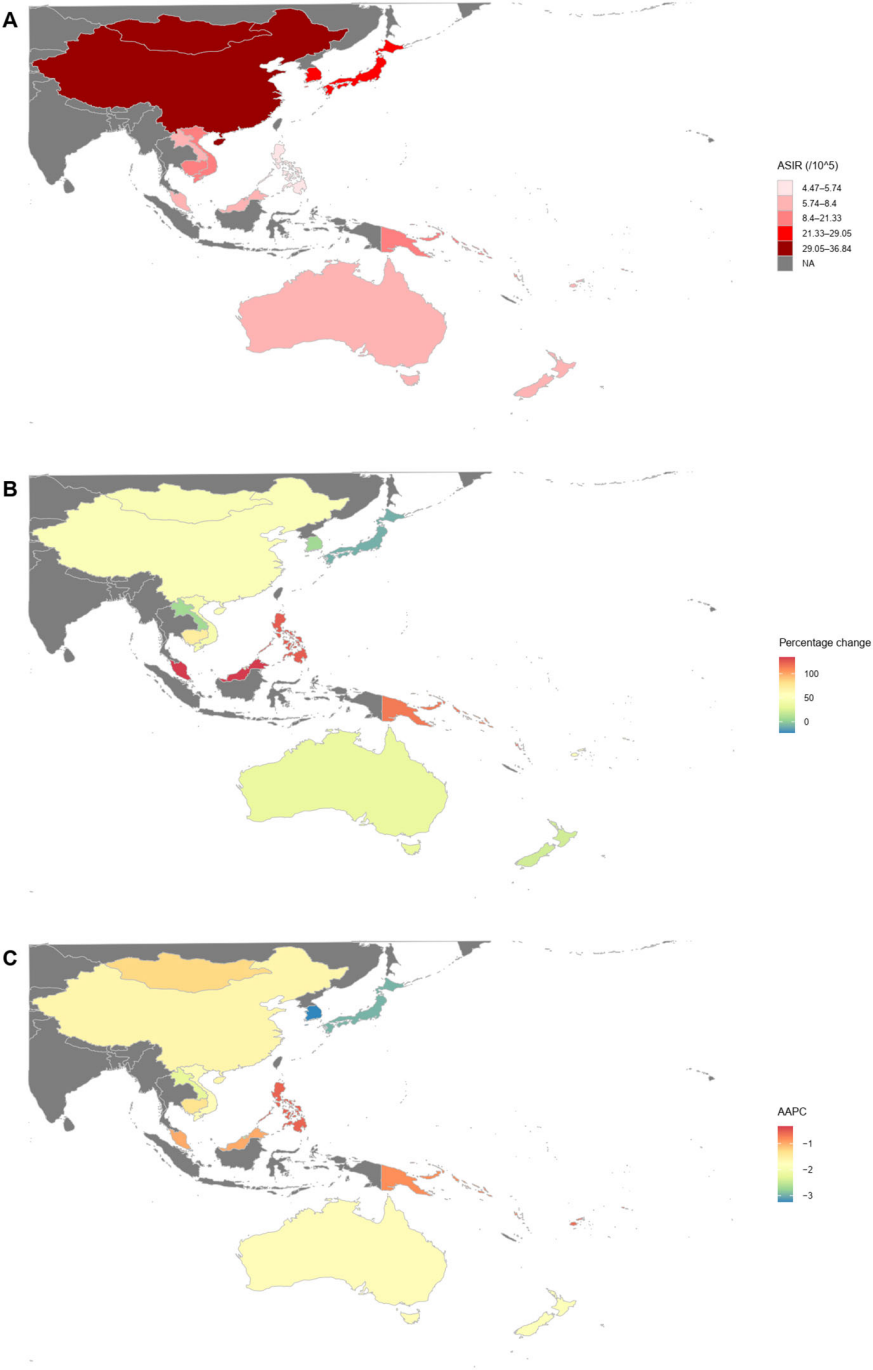
Multi-metric analysis of GC incident cases and ASIR in the countries and territories of the Western Pacific. **(A)** ASIR in 2021; **(B)** Percentage change in the number of GC incident cases from 1990 to 2021; **(C)** AAPC in ASIR from 1990 to 2021. ASIR, age-standardized incidence rate; AAPC, average annual percentage change; GC, gastric cancer.

In 2021, the countries and territories in the Western Pacific with the highest number of incident cases were China, Japan, and South Korea, with China reporting the highest number at 611,798.97 (95% UI: 471,965.81–765,562.25), accounting for 80.89% of the total cases in the region (**Table 2**; **Figure 1**). From 1990 to 2021, only Tokelau (-25.74%), Niue (-22.57%), and Japan (-8.54%) showed a declining trend in incident cases, while most other countries and territories experienced an increase, with Malaysia seeing the largest growth at 134.26% (**Table 2**; **Figure 1**). In 2021, China and Mongolia had an ASIR above the regional average, with Mongolia recording the highest ASIR at 36.83 cases per 100,000 population, while the Philippines had the lowest at 4.48 (95% UI: 3.72–5.94) per 100,000 population (**Table 2**; **Figure 1**). Notably, from 1990 to 2021, the ASIR declined in all countries and territories within the Western Pacific, with South Korea experiencing the most significant decrease, having an AAPC of -3.23 (95% CI: -3.47 to -2.98), whereas Kiribati had the smallest decline, with an AAPC of -0.34 (95% CI: -0.38 to -0.29) (**Table 2**; **Figure 1**).

**Table 2:** Trends in incident cases and ASIR of GC in Western Pacific region countries and territories from 1990 to 2021.

Location	Incident cases			ASIR		
	1990 (95% UI)	2021 (95% UI)	Percentage Change	1990 (95% UI)	2021 (95% UI)	AAPC (95% CI)
Western Pacific Region	551554.15 (476821.81–622213.73)	756302.53 (613600.98–917865.56)	37.12%	47.88 (41.93–54.09)	26.76 (21.71–32.33)	-1.88 (-1.99 to -1.76)
China	407471.29 (337565.45–477568.58)	611798.97 (471965.81–765562.25)	50.15%	48.03 (40.21–56.69)	29.05 (22.42–36.20)	-1.61 (-1.73 to -1.48)
Cambodia	748.82 (560.98–950.24)	1288.42 (949.66–1685.07)	72.06%	16.06 (12.04–20.46)	10.47 (7.96–13.50)	-1.37 (-1.43 to -1.31)
Laos	363.02 (255.24–480.39)	389.32 (286.93–505.17)	7.24%	16.87 (11.98–22.19)	8.40 (6.23–10.87)	-2.22 (-2.30 to -2.15)
Malaysia	807.48 (677.66–955.07)	1891.59 (1613.77–2303.73)	134.26%	8.74 (7.28–10.26)	6.79 (5.78–8.15)	-0.99 (-1.12 to -0.85)
Philippines	1662.75 (1427.68–1989.61)	3734.30 (3086.17–4990.71)	124.59%	5.45 (4.70–6.63)	4.48 (3.72–5.94)	-0.56 (-0.65 to -0.48)
Vietnam	6061.56 (4537.08–7706.20)	8776.58 (6719.67–11482.93)	44.79%	14.85 (11.19–18.85)	8.64 (6.81–11.31)	-1.73 (-1.85 to -1.60)
Fiji	37.07 (19.97–46.10)	58.14 (27.28–79.86)	56.84%	10.18 (5.50–12.48)	8.16 (3.87–10.99)	-0.62 (-0.84 to -0.40)
Kiribati	9.05 (6.94–11.06)	15.59 (11.27–20.07)	72.19%	23.69 (18.31–28.81)	21.33 (15.53–26.86)	-0.34 (-0.38 to -0.29)
Marshall Islands	3.88 (3.06–4.93)	6.37 (4.68–8.21)	64.02%	22.69 (18.05–28.40)	17.73 (13.41–22.43)	-0.79 (-0.86 to -0.72)
Micronesia	11.23 (8.84–14.59)	12.49 (9.28–16.69)	11.28%	22.54 (17.79–29.33)	17.13 (13.16–22.24)	-0.88 (-0.95 to -0.81)
Papua New Guinea	329.55 (218.42–448.41)	707.48 (511.36–927.54)	114.68%	17.94 (11.97–24.22)	13.82 (10.11–17.95)	-0.83 (-0.92 to -0.73)
Samoa	10.70 (7.48–12.94)	15.51 (10.84–19.51)	44.88%	12.81 (9.00–15.39)	10.83 (7.59–13.60)	-0.54 (-0.58 to -0.50)
Solomon Islands	33.20 (21.47–44.92)	70.36 (51.98–94.11)	111.91%	23.85 (16.35–31.21)	19.24 (14.63–25.02)	-0.74 (-0.91 to -0.57)
Tonga	10.86 (8.95–13.83)	13.59 (10.71–17.76)	25.18%	20.16 (16.65–25.65)	17.09 (13.47–22.23)	-0.53 (-0.76 to -0.30)
Vanuatu	12.79 (9.19–17.07)	28.17 (22.09–35.92)	120.29%	20.01 (14.59–26.54)	15.99 (12.71–20.16)	-0.73 (-0.87 to -0.60)
Mongolia	573.80 (466.49–714.52)	848.27 (680.25–1062.33)	47.83%	54.22 (44.05–67.94)	36.83 (29.39–45.27)	-1.26 (-1.60 to -0.92)
Brunei	29.47 (21.71–35.43)	44.80 (36.49–54.37)	52.03%	27.23 (20.02–32.60)	12.96 (10.63–15.43)	-2.44 (-2.90 to -1.97)
Japan	108285.88 (103063.87–111463.19)	99035.03 (85266.05–106694.48)	-8.54%	64.05 (60.74–66.00)	25.54 (23.04–26.96)	-2.95 (-3.16 to -2.74)
South Korea	22166.58 (16776.63–25106.88)	23663.63 (19710.06–29807.13)	6.75%	71.18 (55.99–80.71)	25.76 (21.53–32.40)	-3.23 (-3.47 to -2.98)
Singapore	509.68 (477.39–540.12)	708.04 (634.05–773.66)	38.92%	23.10 (21.63–24.49)	8.47 (7.58–9.26)	-3.11 (-3.48 to -2.75)
Australia	1933.13 (1799.07–2054.02)	2607.70 (2296.19–2870.70)	34.89%	9.88 (9.19–10.49)	5.74 (5.14–6.30)	-1.72 (-1.93 to -1.51)
New Zealand	475.48 (443.28–506.27)	579.33 (518.50–637.67)	21.84%	12.14 (11.29–12.90)	6.93 (6.21–7.61)	-1.84 (-2.56 to -1.11)
American Samoa	4.64 (3.97–5.94)	8.19 (6.76–10.26)	76.44%	20.58 (17.88–26.03)	17.99 (14.80–22.52)	-0.37 (-0.62 to -0.12)
Cook Islands	1.40 (1.15–1.77)	1.75 (1.39–2.18)	24.89%	11.60 (9.61–14.50)	7.02 (5.55–8.84)	-1.62 (-1.75 to -1.49)
Guam	6.55 (5.71–8.14)	11.76 (9.55–13.91)	79.41%	9.27 (8.02–11.18)	5.77 (4.68–6.80)	-1.55 (-2.04 to -1.06)
Nauru	1.27 (0.96–1.69)	1.29 (0.94–1.65)	1.28%	26.31 (20.28–34.59)	21.09 (15.77–26.58)	-0.72 (-0.79 to -0.65)
Niue	0.32 (0.26–0.40)	0.25 (0.20–0.31)	-22.57%	14.52 (11.67–18.21)	11.99 (9.59–14.81)	-0.60 (-0.68 to -0.52)
Northern Mariana Islands	3.29 (2.47–4.41)	6.85 (5.73–8.26)	108.06%	17.54 (13.96–22.24)	14.40 (12.01–17.08)	-0.70 (-0.97 to -0.43)
Palau	2.44 (1.90–3.09)	4.06 (3.20–4.99)	66.37%	24.97 (19.90–31.63)	19.65 (15.88–23.99)	-0.81 (-0.92 to -0.71)
Tokelau	0.22 (0.17–0.28)	0.17 (0.13–0.21)	-25.74%	16.96 (13.11–21.39)	11.23 (8.84–14.29)	-1.31 (-1.37 to -1.26)
Tuvalu	1.44 (1.12–1.81)	1.52 (1.19–1.87)	5.45%	21.83 (17.19–27.21)	15.03 (11.96–18.26)	-1.20 (-1.23 to -1.16)

ASIR, age-standardized incidence rate; GC, gastric cancer; UI, uncertainty interval; AAPC, average annual percent change; CI, confidence interval.

In 2021, the Western Pacific countries and territories with the highest number of GC deaths were China, Japan, and South Korea, with China recording the highest death toll at 445,012.65 (95% UI: 344,736.20–555,833.96) (**Supplementary Table 3**, **Supplementary Figure 1**). From 1990 to 2021, only four countries and territories in the region saw a decline in GC deaths: Nauru (-2.36%), Niue (-24.54%), South Korea (-28.83%), and Tokelau (-29.48%), while most others experienced an increase, with the Philippines showing the largest rise at 118.50% (**Supplementary Table 3**, **Supplementary Figure 1**). In 2021, Mongolia had the highest ASMR for GC in the region at 37.40 (95% UI: 29.36–45.86) per 100,000 population, while Australia had the lowest at 3.78 (95% UI: 3.34–4.12) per 100,000 population (**Supplementary Table 3**, **Supplementary Figure 1**). Notably, from 1990 to 2021, ASMR declined across all countries and territories in the Western Pacific, with South Korea experiencing the most significant decrease, with an AAPC of -4.53 (95% CI: -4.81 to -4.24), while Kiribati had the smallest decline, with an AAPC of -0.38 (95% CI: -0.42 to -0.33) (**Supplementary Table 3**, **Supplementary Figure 1**).

In 2021, the countries and territories with the highest DALYs due to GC in the Western Pacific region were China, Japan, and South Korea, with China ranking first at 10,642,126.54 (95% UI: 8,222,106.35–13,383,779.05) DALYs (**Supplementary Table 4**, **Supplementary Figure 2**). From 1990 to 2021, approximately one-third of the countries and territories in this area experienced a decline in DALYs, with South Korea showing the most significant reduction at -46.83%, while Vanuatu recorded the highest increase at 112.55% (**Supplementary Table 4**, **Supplementary Figure 2**). Regarding ASDR, Mongolia had the highest ASDR in the region in 2021, reaching 930.45 (95% UI: 747.52–1157.92) DALYs per 100,000 people (**Supplementary Table 4**, **Supplementary Figure 2**). Notably, from 1990 to 2021, all countries and territories in the Western Pacific saw a downward trend in ASDR, with South Korea experiencing the largest decrease, as reflected by its AAPC of -5.06 (95% CI: -5.32 to -4.79), whereas Kiribati had the smallest decline, with an AAPC of -0.47 (95% CI: -0.52 to -0.42) (**Supplementary Table 4**, **Supplementary Figure 2**).

### 3.3 GC burden in the Western Pacific region and its representative countries and territories from 1990 to 2021

**Figure 2:**
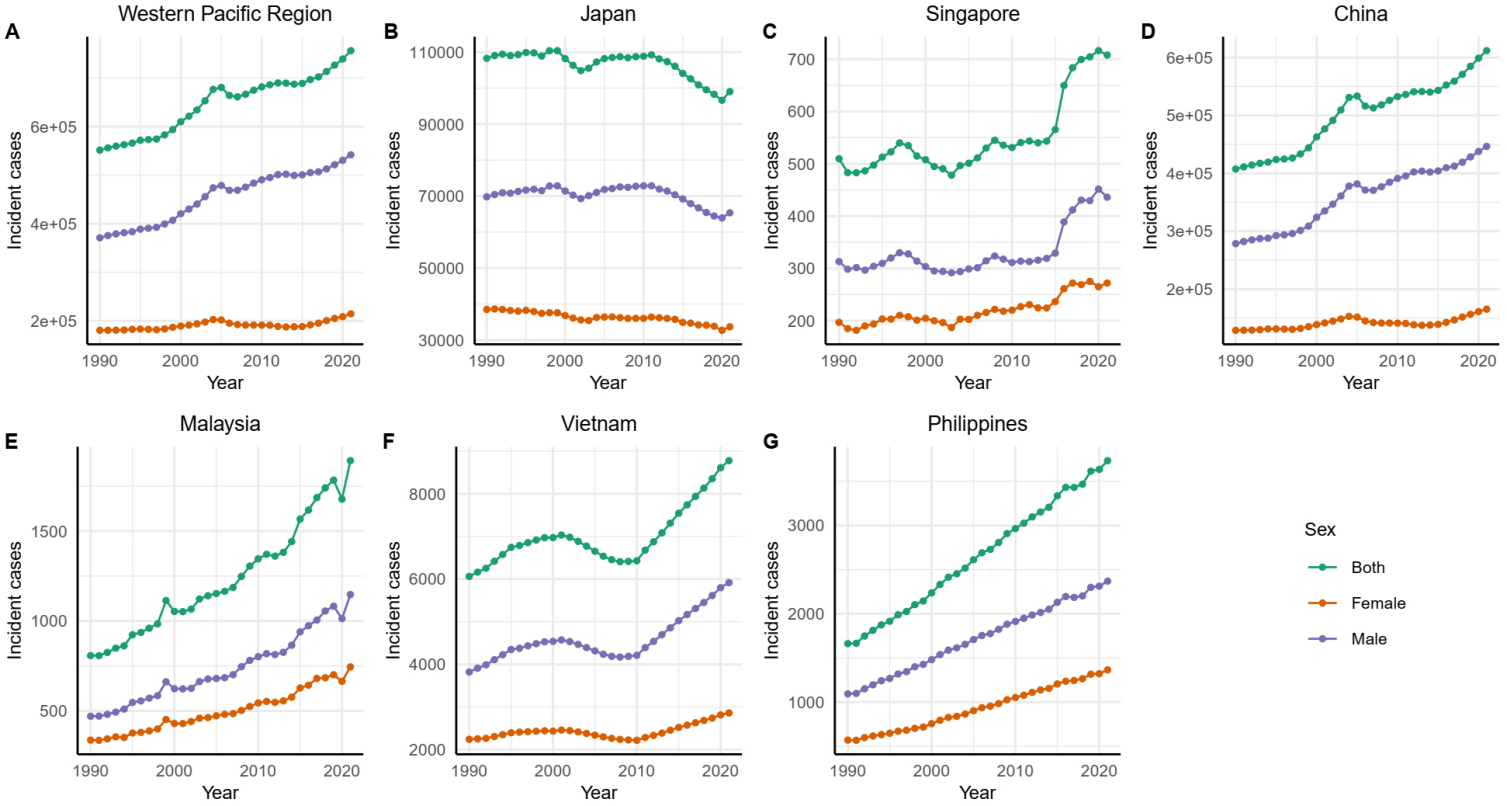
GC incident cases in the Western Pacific region and representative countries and territories from 1990 to 2021. GC, gastric cancer.

Since 1990, the number of incident cases in the Western Pacific region has continued to rise, with China and the Philippines showing an upward trend, while Japan experienced an accelerated decline after 2011, whereas Singapore and Vietnam saw rapid increases starting in 2015 and 2010, respectively (**Figure 2**). In China, the trends of death cases and DALYs showed similar fluctuations (**Supplementary Figures 3**, **4**). In contrast, Japan’s number of deaths remained relatively stable from 1990 to 2021, though DALYs showed a steady decline (**Supplementary Figures 3**, **4**). The Philippines experienced a steady increase in both death cases and DALYs, whereas Singapore saw a sharp rise in both indicators from 2015 onward (**Supplementary Figures 3**, **4**). Similarly, Vietnam also displayed an accelerating growth trend after 2010 (**Supplementary Figures 3**, **4**).

From 1990 to 2021, the ASIR, ASMR, and ASDR of GC in the Western Pacific region and its representative countries showed an overall declining trend (**Supplementary Figures 5**–**7**). The ASIR, ASMR, and ASDR for males were consistently higher than those for females, with both sexes exhibiting similar trends of change (**Supplementary Figures 5**–**7**). The Western Pacific region and China experienced a slight peak around 2005, followed by an overall decline (**Supplementary Figures 5**–**7**). Japan demonstrated a relatively stable downward trend, while Singapore showed a steady decline from 1990 to 2015, followed by a fluctuation of increase and then decrease after 2015 (**Supplementary Figures 5**–**7**). Malaysia exhibited a more fluctuating downward trend (**Supplementary Figures 5**–**7**). Vietnam displayed an overall decline, with an accelerated decrease after 1995 and a slight slowdown around 2010. Similarly, the Philippines showed an overall downward trend, with a noticeable acceleration in the decline after 2005 (**Supplementary Figures 5**–**7**).

### 3.4 GC burden by age and sex in the Western Pacific region in 2021

**Figure 3:**
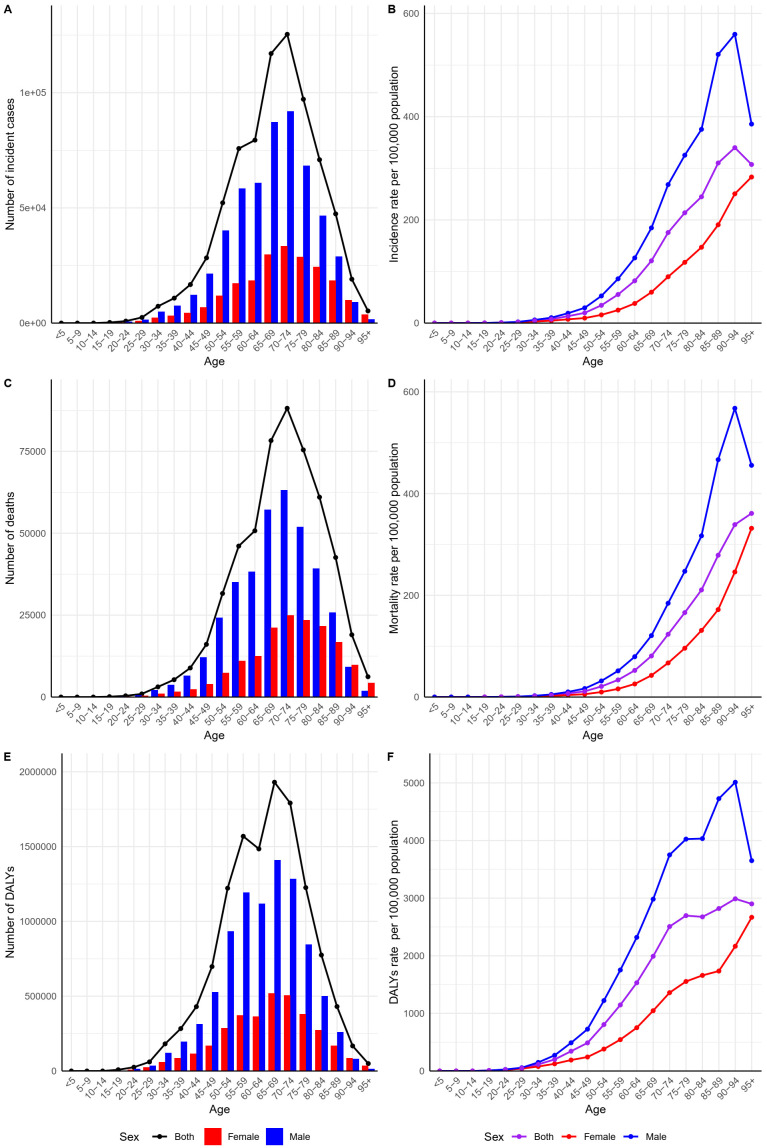
GC burden in the Western Pacific region in 2021 by sex and age group. **(A)** Number of GC incident cases; **(B)** GC incidence rate; **(C)** Number of GC deaths; **(D)** GC mortality rate; **(E)** GC DALYs; **(F)** GC DALY rate. DALYs, disability-adjusted life years; GC, gastric cancer.

In this study, the population of the Western Pacific region was divided into 20 age groups, each spanning 5 years (**Figure 3**). The findings revealed that in most age groups, the number of incident cases, number of deaths, DALYs, and ASRs of GC were consistently higher in males than in females (**Figure 3**). The number of incident cases, number of deaths, and DALYs for both sexes, as well as for males and females separately, exhibited an overall trend of initial increase followed by a decline, with the turning point occurring between the ages of 65 and 74 (**Figures 3A, C, E**). Regarding ASRs, males had the highest rates, followed by the overall population, with females showing the lowest rates (**Figures 3B, D, F**). Among males, the ASRs increased initially and then decreased, with a turning point in the 90-94 age group (**Figures 3B, D, F**). In contrast, females exhibited a generally continuous increase in the ASRs with advancing age (**Figures 3B, D, F**).

### 3.5 Health inequalities

**Figure 4:**
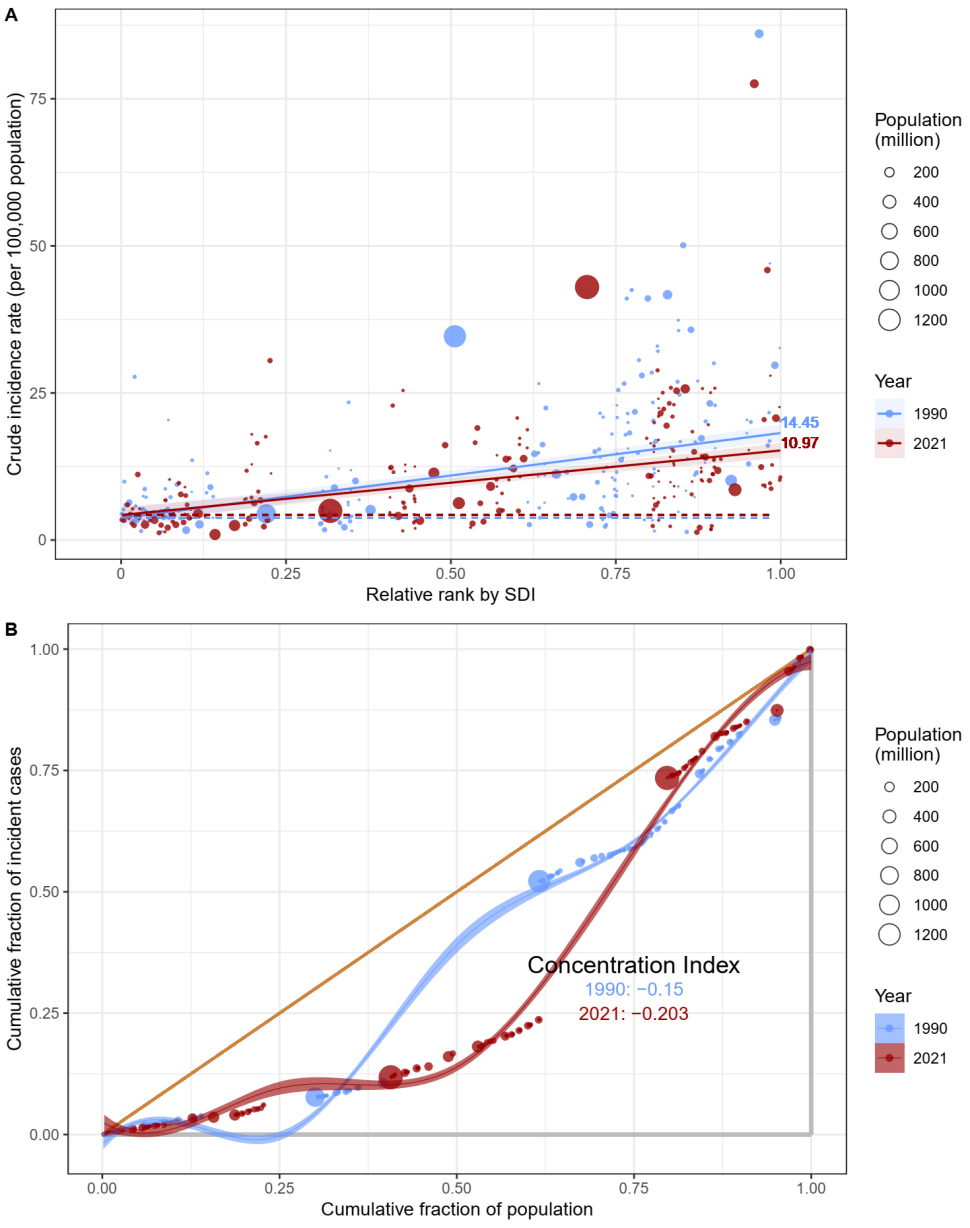
Results of the health inequality analysis. **(A)** Health inequality regression curve for the ASIR of GC; **(B)** Concentration curve for the ASIR of GC. SDI, Socio-demographic index; ASIR, age-standardized incidence rate; GC, gastric cancer.

From 1990 to 2021, the gap in the overall incidence, mortality, and DALYs rates of GC between countries with the highest and lowest SDI narrowed. Specifically, the Slope SII for incidence decreased from 14.45 to 10.97 (**Figure 4A**), for mortality from 12.89 to 8.51 (**Supplementary Figure 8A**), and for DALYs rate from 265.69 to 134.13 (**Supplementary Figure 9A**).

However, in contrast, the relative gradient of inequality, as measured by the Concentration Index, exhibited a different trend. In 1990, the Concentration Index for incidence, mortality, and DALYs rates was -0.15, -0.09, and -0.05, respectively (**Figure 4B**; **Supplementary Figures 8B**, **9B**). By 2021, these values had changed to -0.203, -0.154, and -0.108, indicating an increase in the relative degree of inequality (**Figure 4B**; **Supplementary Figures 8B**, **9B**).

### 3.6 BAPC prediction

**Figure 5:**
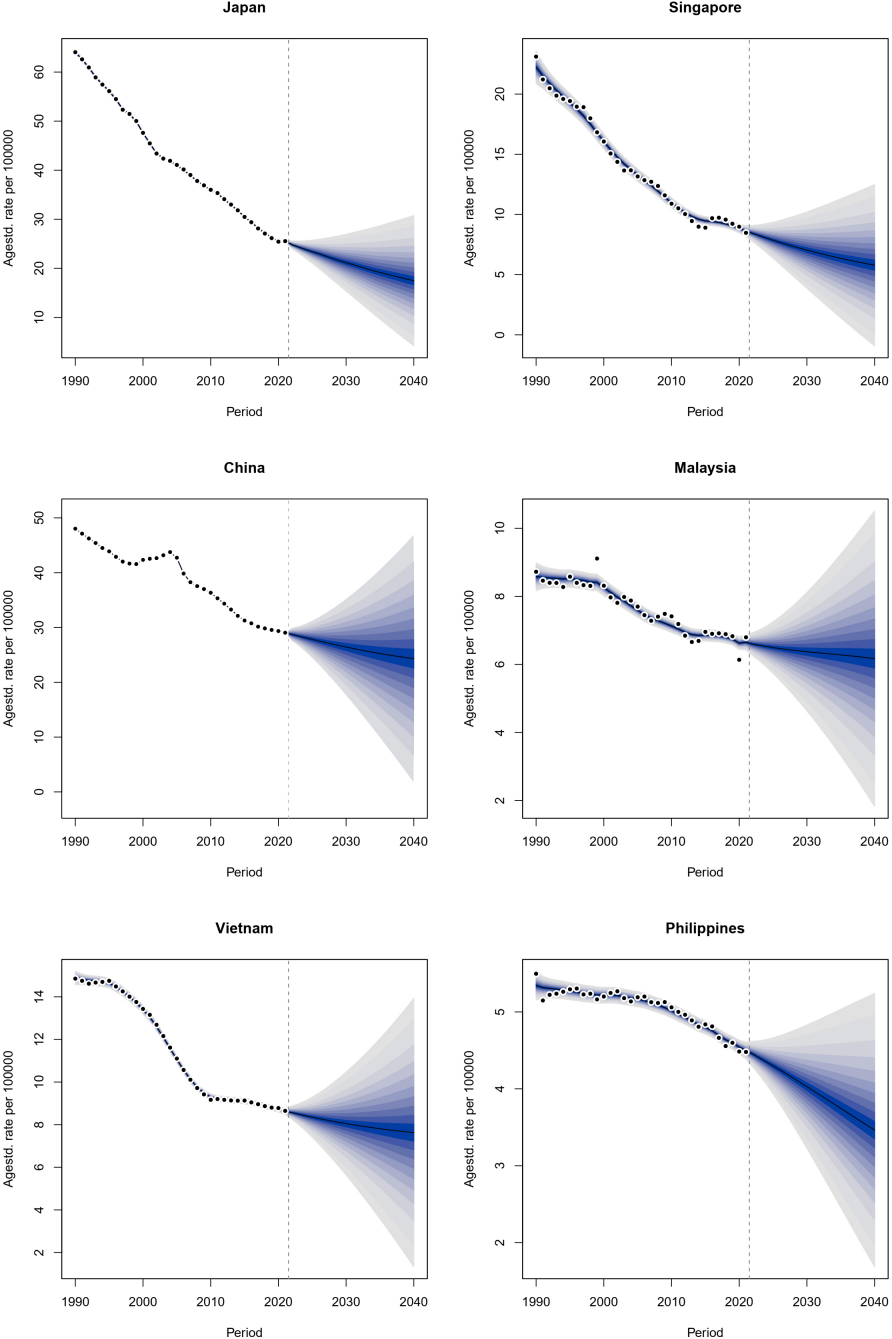
Projected ASIR in six countries of the Western Pacific region by 2040. Agestd. rate, age-standardized rate; ASIR, age-standardized incidence rate.

The study selected representative countries for forecasting ASRs through 2040. Japan and Singapore were chosen to represent high-income countries, China and Malaysia to represent upper-middle-income countries, and Vietnam and the Philippines to represent lower-middle-income countries. The ASIR, ASMR, and ASDR for GC in these six countries were predicted using the BAPC model. The projections indicate an upward trend for all indicators except for the ASDR in Malaysia, which is expected to decrease by 2040 (**Figure 5**; **Supplementary Figures 9**, **10**).

## 4 Discussion

This study, based on GBD 2021 data, analyzes the burden of GC in the Western Pacific from 1990 to 2021. The results show an increase in incident cases and deaths, while DALYs have declined. The region has the highest ASRs among the six WHO regions, exceeding the global average. However, its ASR decline surpasses the global trend, indicating a notable improvement in disease burden. Males bear a significantly higher burden than females. While economically developed areas experience a heavier burden, health inequalities are narrowing, whereas low-income populations face worsening health conditions. Projections suggest a continued decline in ASRs in the region.

The study indicates that although the ASRs of GC in the Western Pacific region were higher than the global level in 2021, the decline in ASRs in the region exceeded the global average. Previous studies suggest that the high prevalence of Helicobacter pylori infection and the extensive consumption of traditionally salted and smoked foods are major risk factors contributing to the elevated GC incidence in this region (34–36). As sanitary conditions improve and antibiotic use becomes widespread, the incidence of Helicobacter pylori infection is gradually declining, leading to a decrease in GC rates (4). Traditionally, some countries and regions in the Western Pacific (such as Japan, South Korea, and China) have diets high in salt-preserved and smoked foods, which are associated with an increased risk of GC. With a growing awareness of health, dietary habits are gradually improving, characterized by increased intake of fresh vegetables and fruits, and reduced salt in processed foods, consequently lowering the risk of GC (14). As the public’s awareness of health prevention rises, especially in countries like Japan and South Korea, early screening for GC (such as endoscopy) has become a routine part of health check-ups (37). Early detection significantly enhances treatment outcomes and reduces mortality rates. The promotion of such screening methods has effectively decreased late-stage GC cases and associated mortality. Advances in medical technology have also improved treatment options for GC, including minimally invasive surgery, targeted therapy, and immunotherapy, all of which enhance patient survival rates and reduce mortality related to GC. However, the Western Pacific region experienced substantial population growth and aging from 1990 to 2021; although the ASRs show a declining trend, the increase in the absolute number of new cases and deaths persists due to the growing population base.

Particular attention should be given to China, a major contributor to the GC burden in the Western Pacific and globally. In 2021, China ranked first in new GC cases, deaths, and DALYs in the Western Pacific, accounting for over half of the total burden. Its ASIR, ASMR, and ASDR ranked second, third, and fifth in the Western Pacific region, respectively. This is largely due to China’s large population and rapid growth in recent years, alongside poor dietary and lifestyle habits. Studies show that since 2012, alcohol consumption in China has surpassed the global average, while smoking rates and high salt diets remain significantly elevated (11, 38, 39). Additionally, the high prevalence of Helicobacter pylori infection is a critical factor contributing to the rising incidence (40).

Conversely, South Korea and Japan, traditional high-incidence areas for GC, exhibit high consumption of preserved and smoked foods, coupled with elevated Helicobacter pylori infection rates. However, they benefit from government-funded health initiatives and heightened health awareness, leading to high participation in GC screening programs. In both Japan and South Korea, active GC screening programs have been implemented for populations aged 40 and older (11). In Japan, GC screening began in the 1960s with barium swallow tests and eventually transitioned to endoscopy (37). In South Korea, GC screening is provided either for free or at only 10% of the cost, ensuring that all eligible individuals are covered at minimal or no expense (37). Although ASRs of GC in China are declining, they are not as favorable as those in South Korea and Japan. China’s screening programs have not been widely implemented nationwide, being mainly concentrated in high-incidence areas and among high-risk populations, with uneven distribution of resources between urban and rural areas (41). However, the “Healthy China 2030” initiative offers hope for enhanced GC screening in the future (42).

The study revealed that, across nearly all age groups, the burden of GC is greater in males than in females. While extensive research supports this phenomenon, the specific mechanisms remain poorly understood (11, 43). Further investigation is essential to identify the underlying causes and develop targeted preventive measures. We speculate that factors such as genetics and lifestyle choices, particularly higher rates of smoking and alcohol consumption in males, may play a role (44, 45). Additionally, some studies suggest that estrogen may exert a protective effect against GC, potentially contributing to the lower incidence rates observed in females (11).

Health inequality analysis reveals that although disparities in GC between countries, as measured by the SDI, have narrowed, the disease burden is increasingly concentrated among impoverished populations. The prevalence of Helicobacter pylori infection is strongly linked to a country’s socioeconomic status and access to universal healthcare (11, 46, 47). Poorer regions often experience higher rates of Helicobacter pylori infection and lack adequate healthcare resources, leading to insufficient treatment. While endoscopy remains the gold standard for GC screening, its high cost and limited healthcare infrastructure in certain regions impede widespread screening, severely reducing early diagnosis rates and worsening outcomes for GC patients. Furthermore, our study found that countries experiencing the largest declines in ASRs are predominantly in developed regions (such as Japan and South Korea), suggesting that these factors may also contribute to this trend. This indicates that underdeveloped regions will likewise need more attention and resources in the future.

Addressing gastric cancer disparities requires stronger international collaboration and regional policies, especially in low-SDI countries with limited healthcare resources. Global cooperation can enhance knowledge sharing, healthcare infrastructure, and access to essential services like Helicobacter pylori eradication and cancer screening. Expanding WHO and regional support can help implement cost-effective screening methods, while universal healthcare and financial subsidies can improve early diagnosis and treatment. Collaboration between high- and low-SDI countries can strengthen capacity-building, improve diagnostics, and raise awareness. Leveraging successful strategies from countries like Japan and South Korea can guide tailored solutions. Ultimately, targeted policies and global cooperation are key to reducing the gastric cancer burden in developing regions.

This study utilizes the most comprehensive and up-to-date GBD data to analyze GC burden trends in the Western Pacific region; however, several limitations exist. As a retrospective analysis based on GBD 2021, its reliability depends on data accuracy, which varies across countries. In developing nations, incomplete diagnostic methods and data collection systems may introduce bias, while limited coverage of civil registration and vital statistics systems and gaps in health surveys and hospital reports increase uncertainty in disease burden estimates. Additionally, in regions with poor data availability, GBD modeling relies on interpolated data that may not accurately reflect local conditions, affecting trend analyses. While regional studies suggest significant epidemiological differences between cardia and non-cardia GC, the GBD database lacks specific data for further exploration. Although our predictions offer valuable insights into future trends, the GBD forecasting model is based on historical data extrapolation and does not fully account for evolving healthcare systems and policy changes, introducing uncertainty. Furthermore, variations in pathological staging and treatment strategies impact prognosis, but data limitations prevented a more in-depth analysis. Future research should strengthen large-scale epidemiological studies, integrate multiple data sources to validate trends, and improve data quality through standardized and comprehensive collection. Additionally, expanding disease-specific information in the GBD database would enhance the accuracy and comparability of analyses.

## 5 Conclusion

Although the burden of GC in the Western Pacific region remains higher than the global average, the rate of reduction in this region has surpassed the global trend, and health inequalities are gradually decreasing. However, despite the reduction in health disparities, GC cases are increasingly concentrated in less developed areas. The burden of GC is significantly higher in men than in women, and this issue should receive more attention in the future.

## Data Availability

The original contributions presented in the study are included in the article/**Supplementary Material**. Further inquiries can be directed to the corresponding author/s.
