# Supplementary material for: Trends in gastric cancer burden in the Western Pacific region from 1990 to 2021 and projections to 2040
Source: Front Oncol. 2025 Mar 12;15:1506479. doi: 10.3389/fonc.2025.1506479 (PMC11936811; doi:10.3389/fonc.2025.1506479)
Supplement: Supplementary file 2 [file DataSheet1.docx]

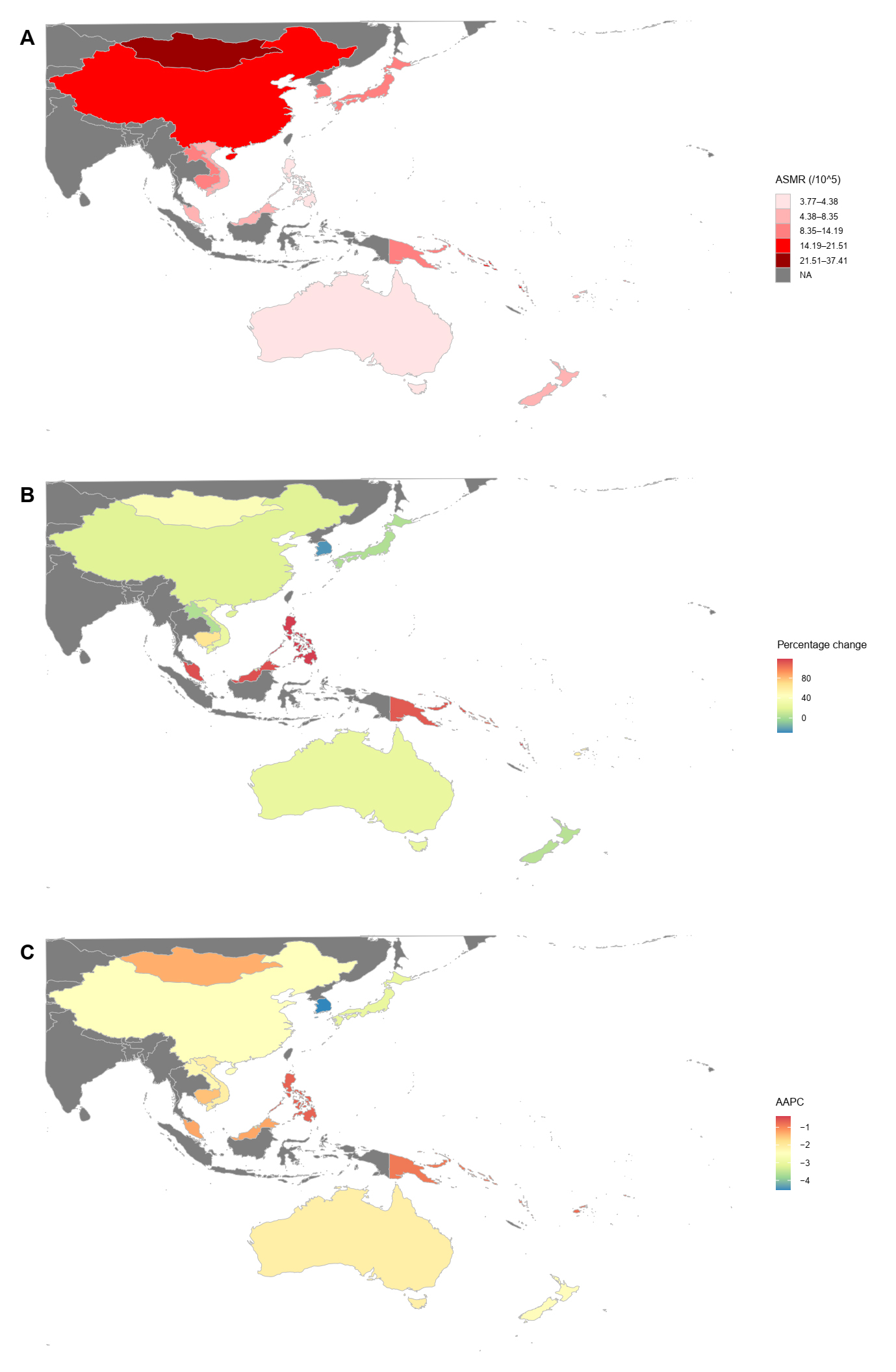


Supplementary Figure 1: Multi-metric analysis of GC deaths and ASMR in the countries and territories of the Western Pacific. A. ASMR in 2021; B. Percentage change in the number of GC deaths from 1990 to 2021; C. AAPC in ASMR from 1990 to 2021. ASMR: age-standardized mortality rate; AAPC: average annual percentage change; GC: gastric cancer.


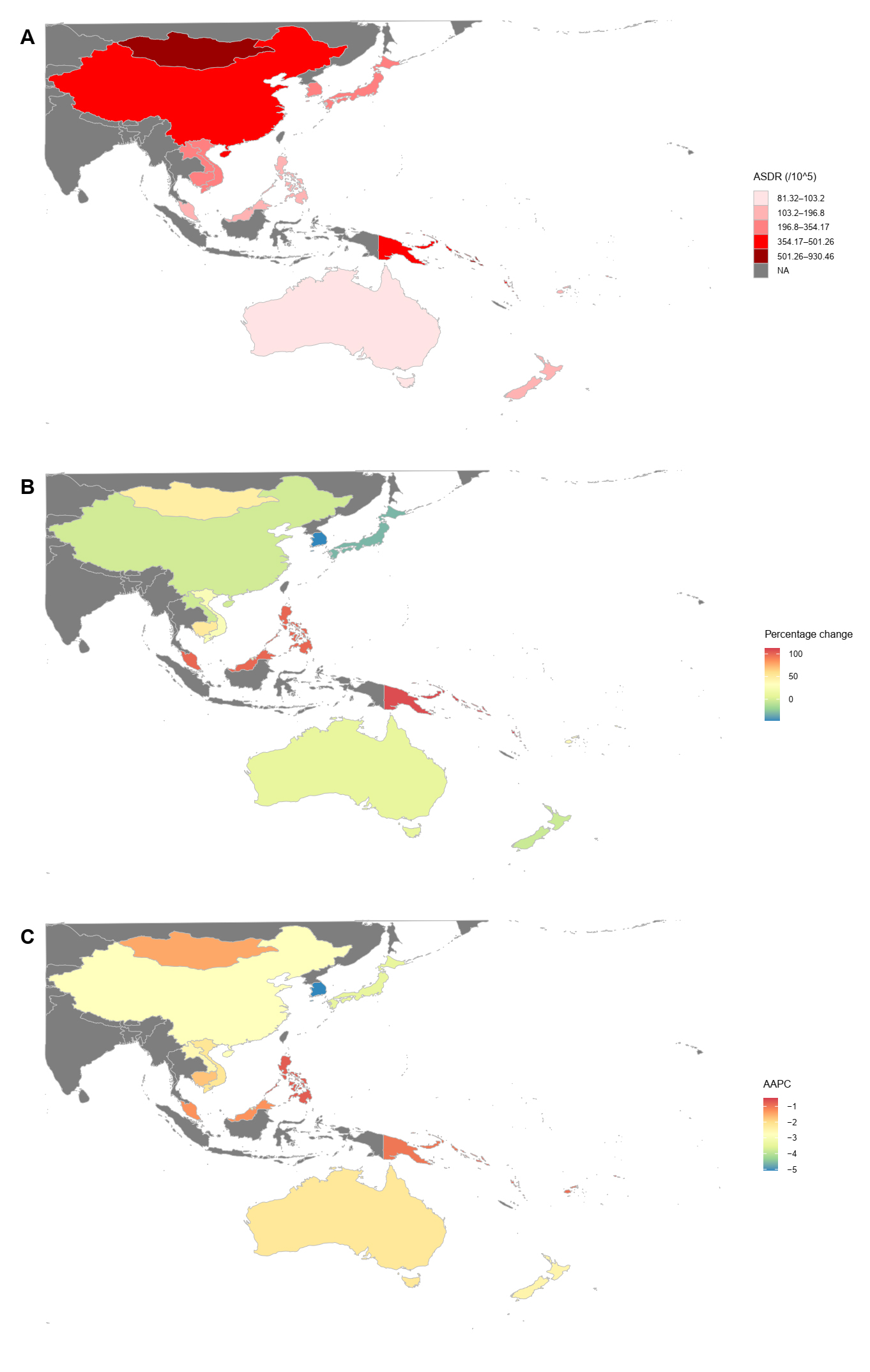


Supplementary Figure 2: Multi-metric analysis of GC DALYs and ASDR in the countries and territories of the Western Pacific. A. ASDR in 2021; B. Percentage change in the number of GC DALYs from 1990 to 2021; C. AAPC in ASDR from 1990 to 2021. ASDR: age-standardized DALYs rate; AAPC: average annual percentage change; GC: gastric cancer; DALYs, disability-adjusted life years.


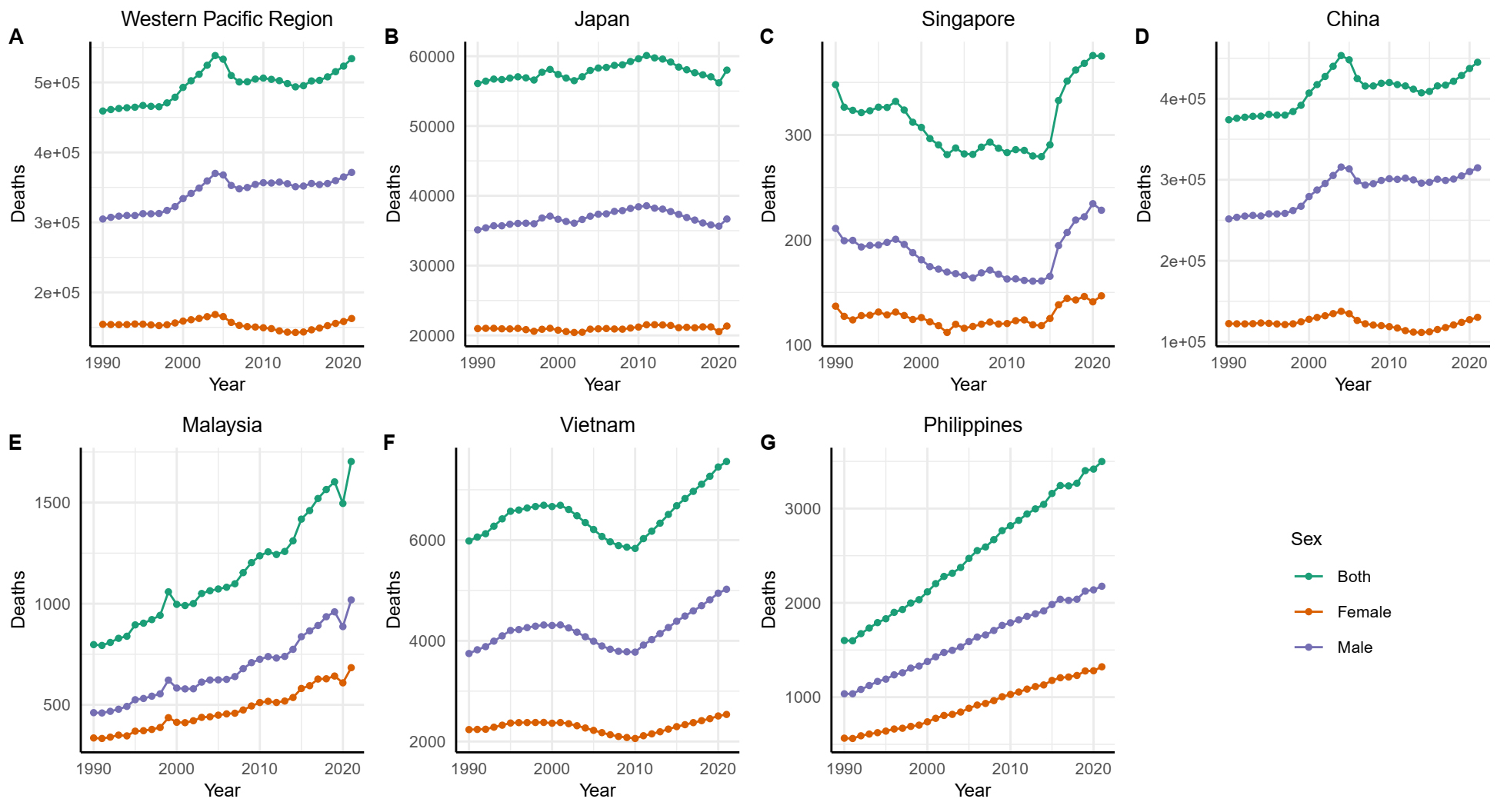


Supplementary Figure 3: GC deaths in the Western Pacific region and representative countries and territories from 1990 to 2021. GC, gastric cancer.


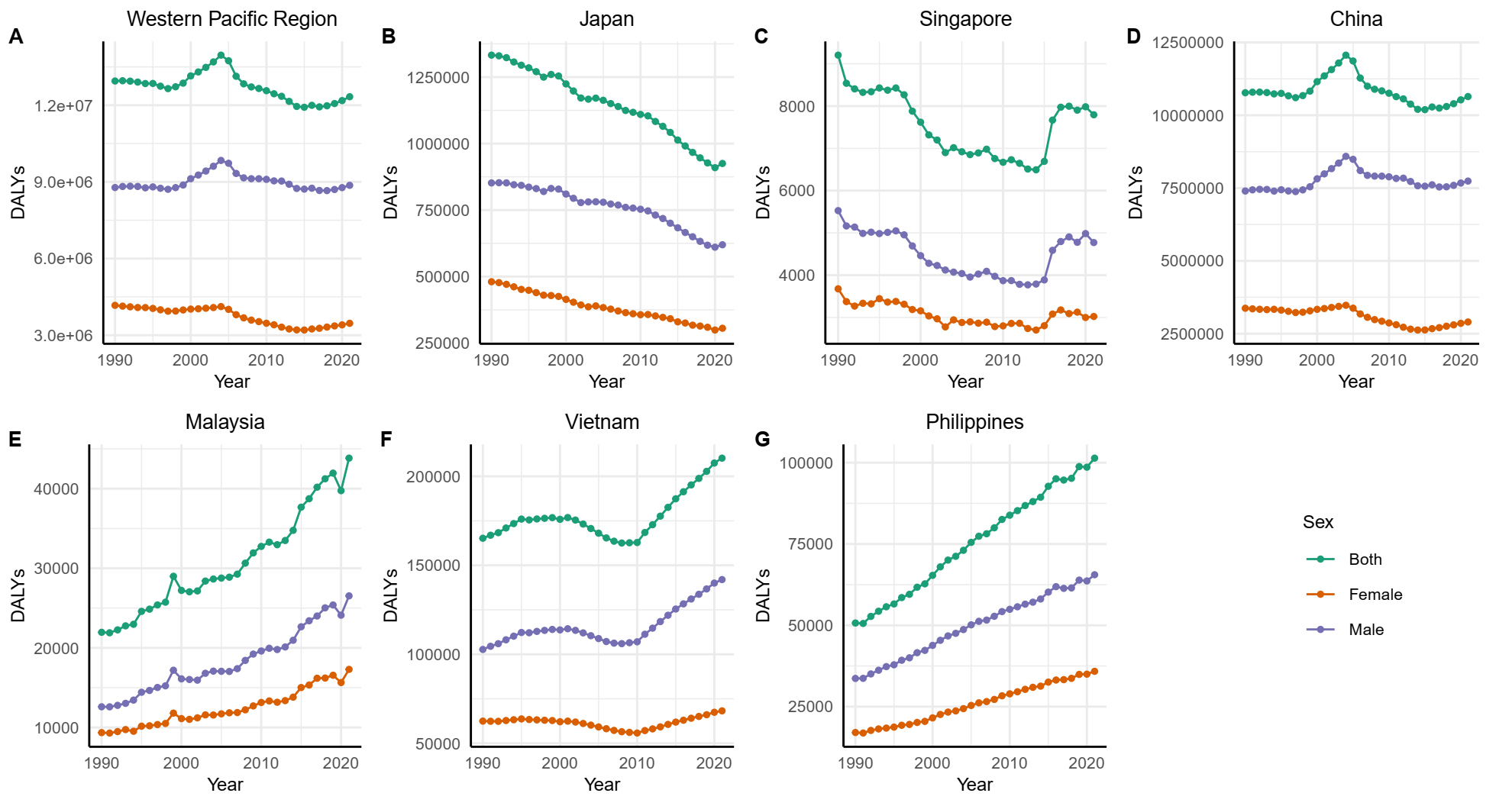


Supplementary Figure 4: GC DALYs in the Western Pacific region and representative countries and territories from 1990 to 2021. DALYs, disability-adjusted life years; GC, gastric cancer.


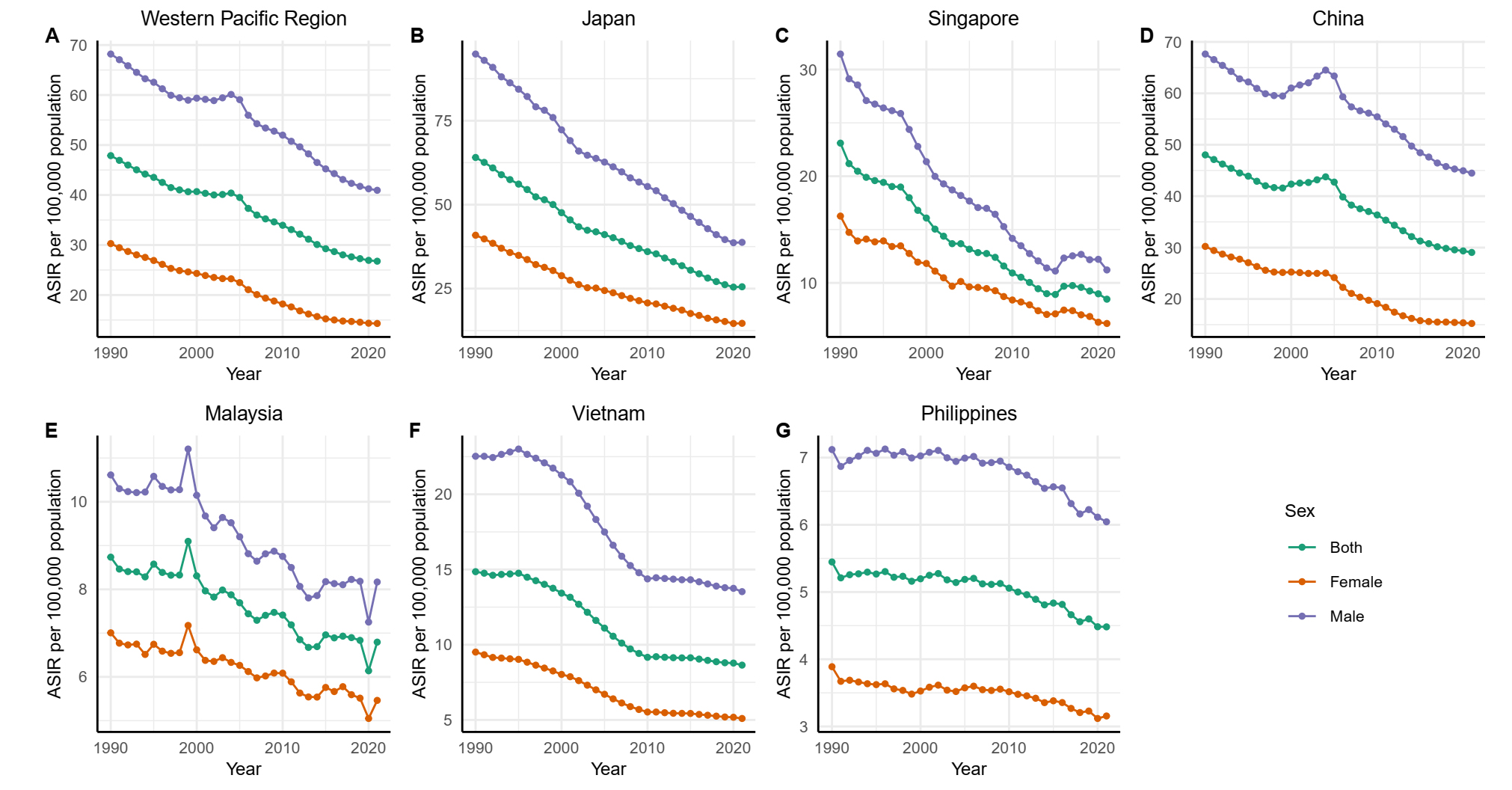


Supplementary Figure 5: GC ASIR in the Western Pacific region and representative countries and territories from 1990 to 2021. ASIR, age-standardized incidence rate; GC, gastric cancer.


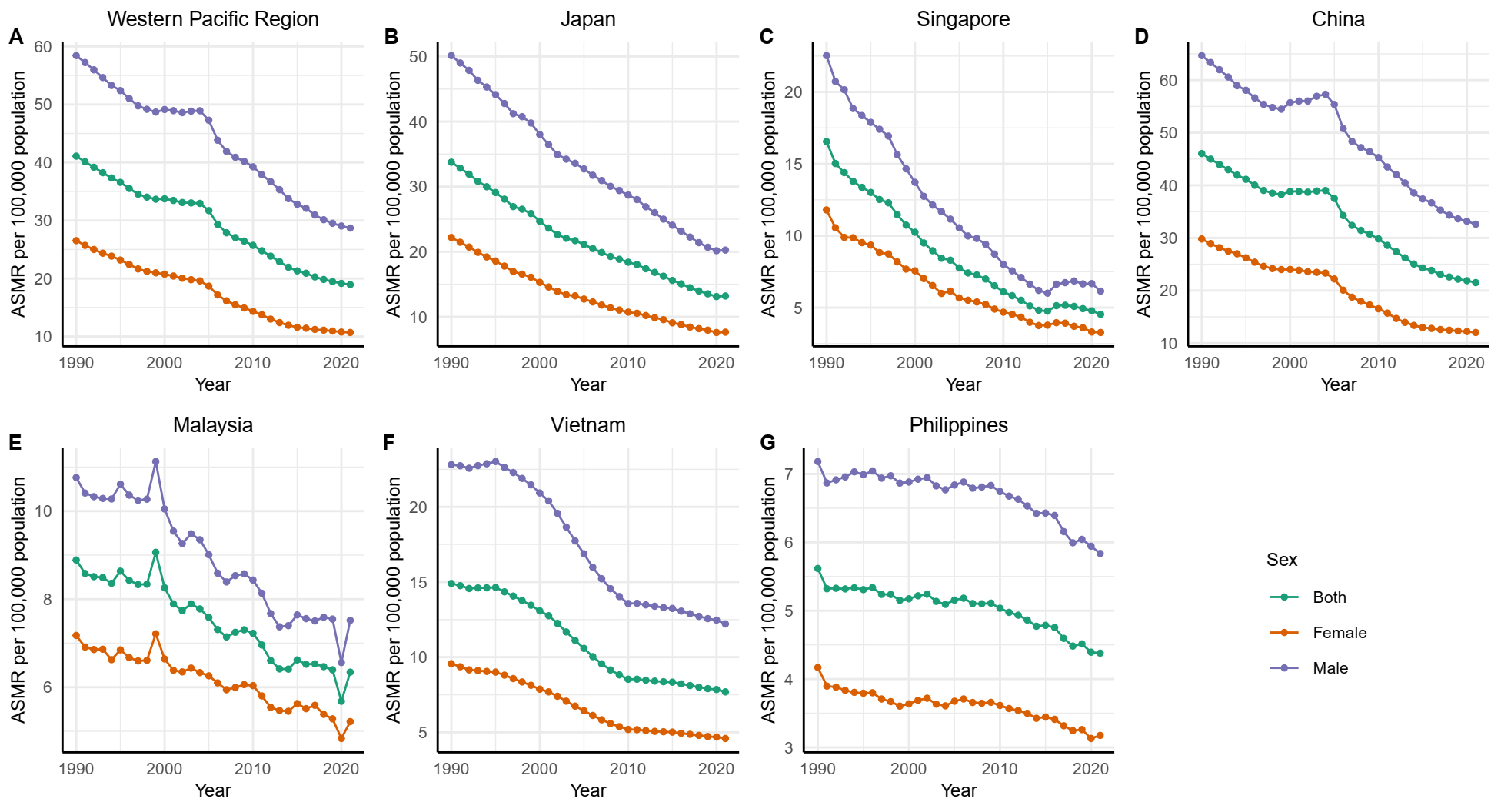


Supplementary Figure 6: GC ASMR in the Western Pacific region and representative countries and territories from 1990 to 2021. ASMR, age-standardized mortality rate; GC, gastric cancer.


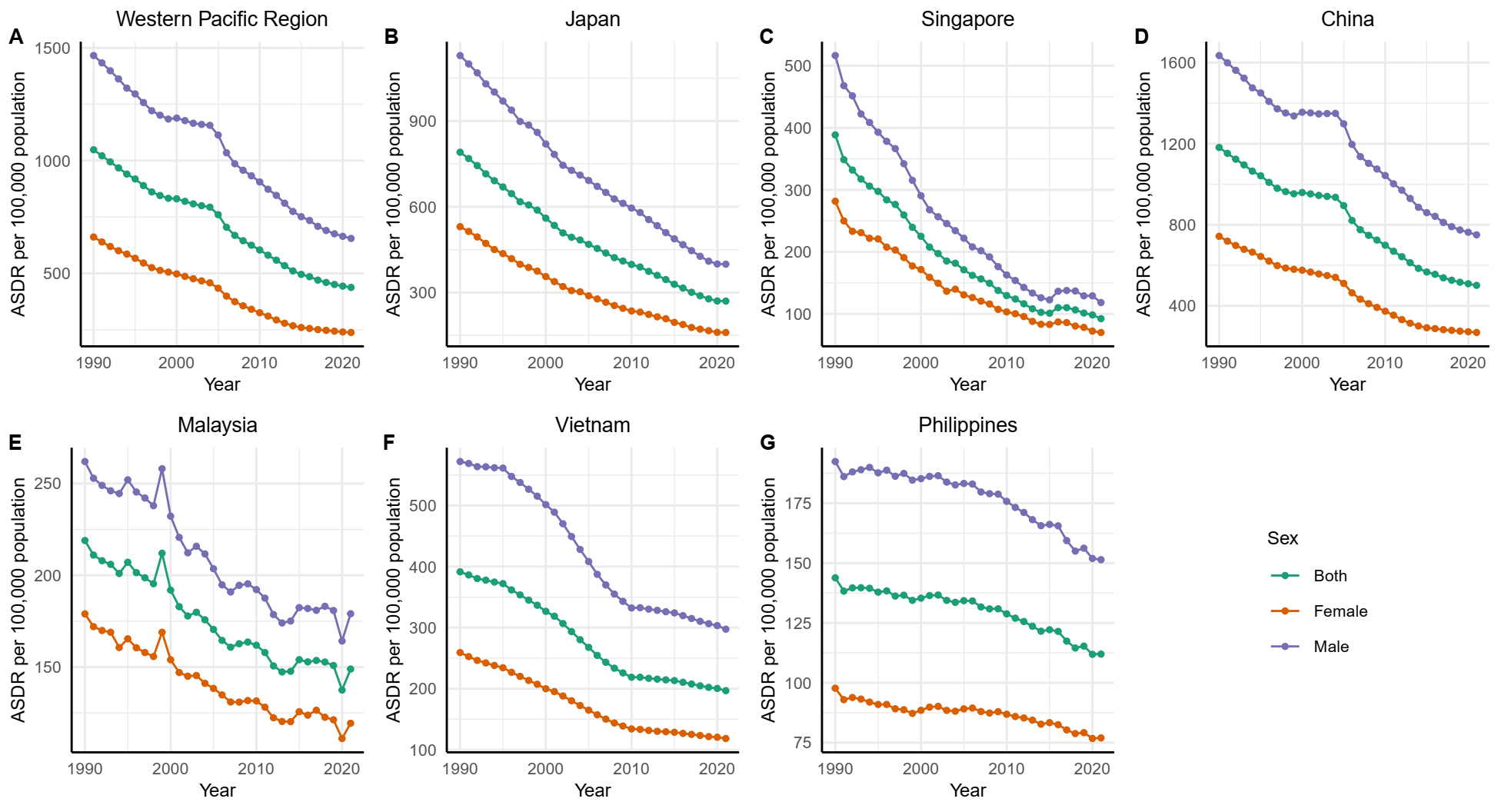


Supplementary Figure 7: GC ASDR in the Western Pacific region and representative countries and territories from 1990 to 2021. ASDR, age-standardized disability-adjusted life years rate; GC, gastric cancer.


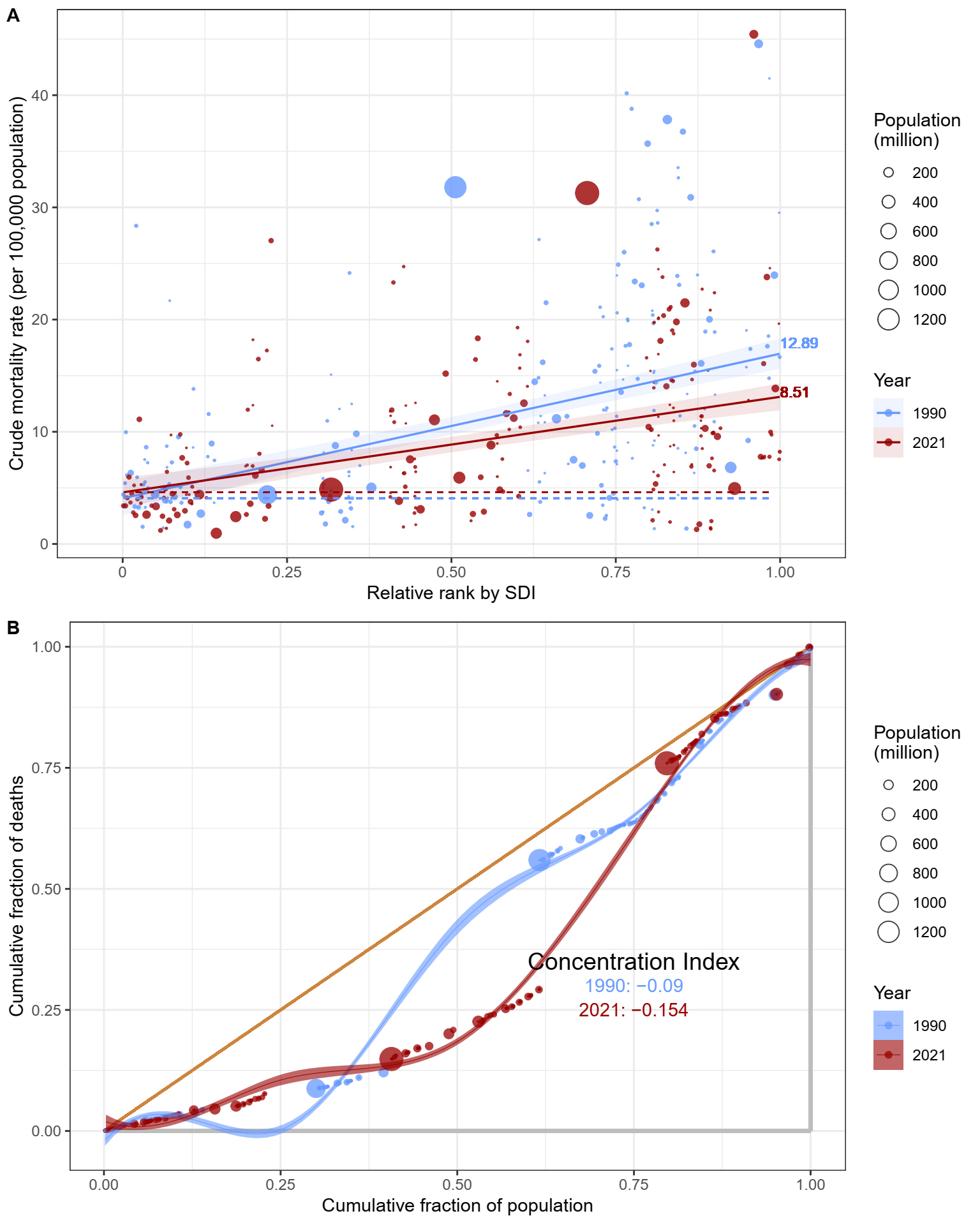


Supplementary Figure 8: Results of health inequality analysis of mortality in the Western Pacific region. SDI, Socio-demographic index.


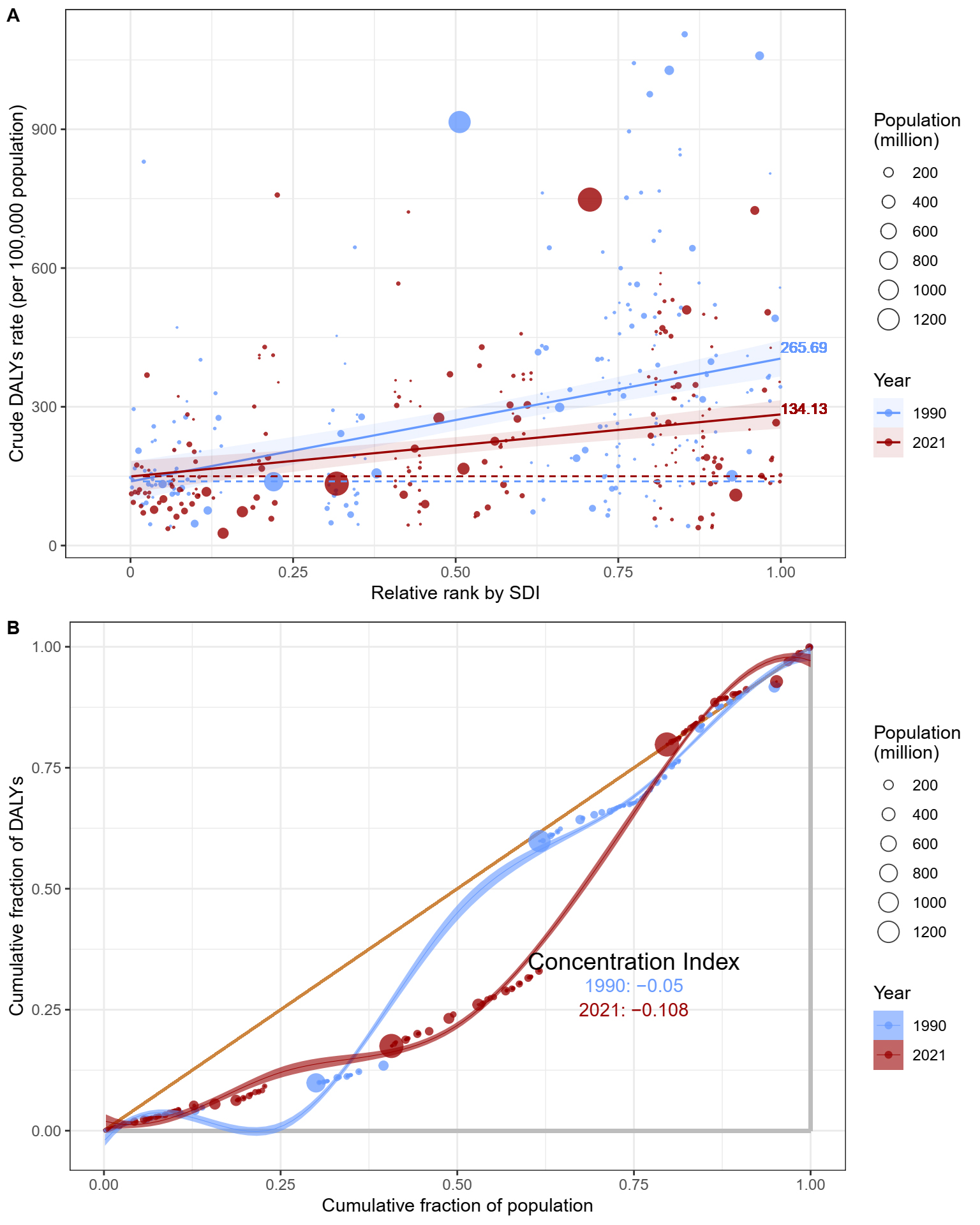


Supplementary Figure 9: Results of health inequality analysis of DALYs in the Western Pacific region. DALYs, disability-adjusted life years; SDI, Socio-demographic index.


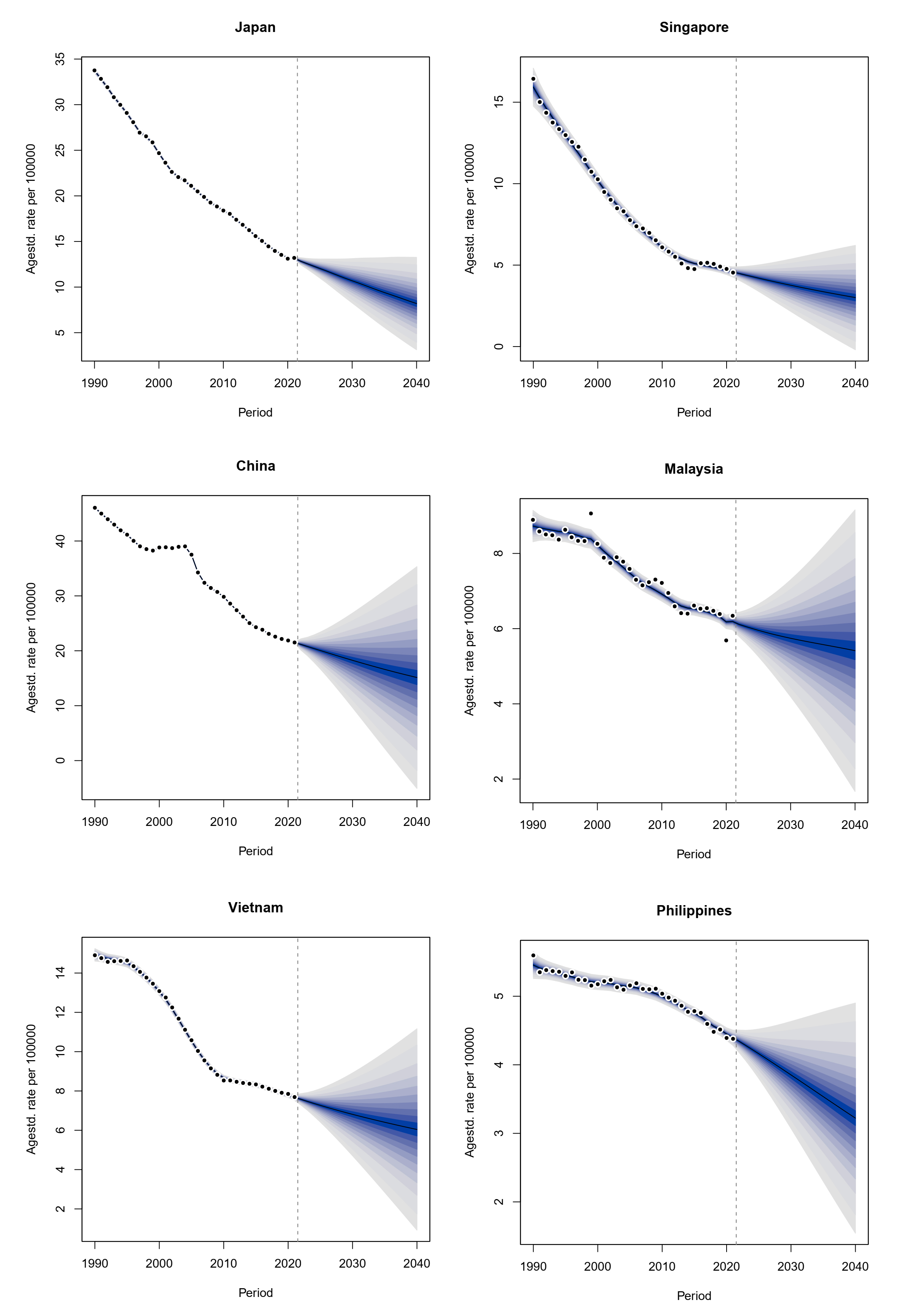


Supplementary Figure 10: Projected ASMR in six countries of the Western Pacific region by 2040. ASMR, age-standardized mortality rate; Agestd. rate, age-standardized rate.


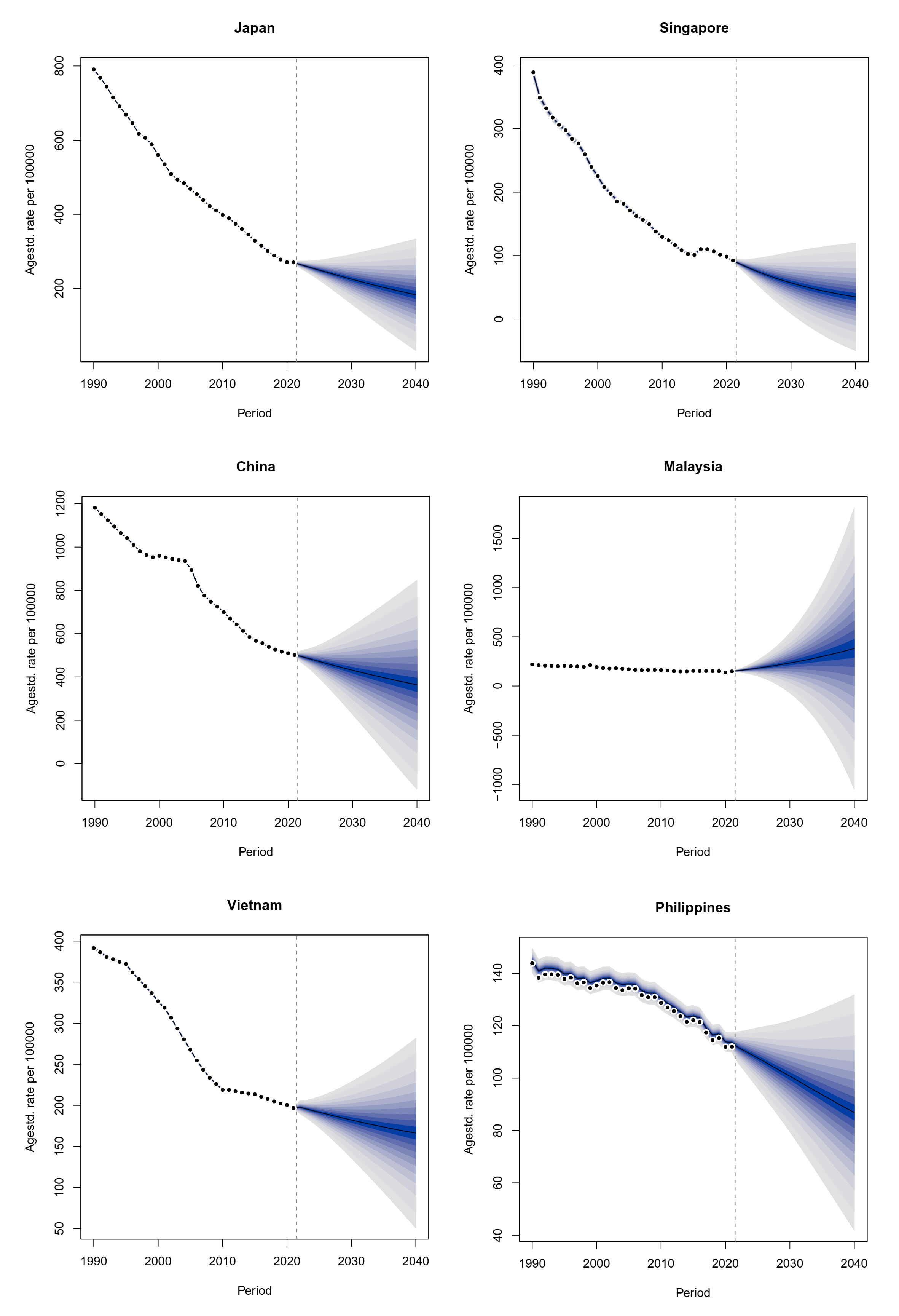


Supplementary Figure 11: Projected ASDR in six countries of the Western Pacific region by 2040. ASMR, age-standardized disability-adjusted life years rate; Agestd. rate, age-standardized rate.
